# Supplementary material for: Detection of COVID-19 in smartphone-based breathing recordings: A pre-screening deep learning tool
Source: PLoS One. 2022 Jan 13;17(1):e0262448. doi: 10.1371/journal.pone.0262448 (PMC8758005; doi:10.1371/journal.pone.0262448)
Supplement: Attachment — Submitted filename: Response to Reviewers.pdf [file pone.0262448.s001.pdf]

REVIEWER 1

In the paper, the authors propose using CNN-BiLSTM for COVID19 detection. The proposal was explained and described. In general, it is a good approach and shows great results. Some of my issues that should be improved.

Concern #1: Discuss in more detail the latest achievements in using machine learning for covid detection/classification.

Response: The authors appreciate all concerns of this reviewer, which have significantly improved the proposed manuscript. We have added a paragraph about machine learning-related literature in COVID-19 detection using CT, X-ray, and Ultrasound imaging (**See Introduction**). More information about current achievements in detecting COVID-19 in breathing/coughing recordings were added as well (**See Results and Table 4, Page 14**) based on **Concern #3**.

Concern #2: Add a pseudocode of your proposal.

Response: We have added a pseudocode for the training procedure of the proposed deep learning model including feature extraction, MFCC transformation, and CNN-BiLSTM deep features extraction (**See Algorithm 1, Page 6**).

Concern #3: Make a comparison with state-of-art.

Response: We have created a summary table to show the current state-of-art studies in detecting COVID-19 in breathing/coughing recordings using machine learning (**See Table 4, Page 14**). We have added a section as well to discuss the performance of our model relative to these studies (**See Results**).

REVIEWER 2

This manuscript is based on handcrafted and deep features. topic of the research is interesting but unfortunately, this manuscript is lack of novelty. The CNN-BiLSTM based features extraction are not as a contribution. Also, the results section is not impressive. I do not recommend to accept this manuscript.

Response: We appreciate the concern of this reviewer about the contributions of the proposed study. This work contributes to the field of research towards developing a purely digital COVID-19 diagnostic test by applying deep learning methods to analyze breathing recordings (shallow and deep breathing). When compared to the current gold-standard PCR test, this tool would be cost-

effective, safe for social distancing protocols, faster in returning results, and does not require experts nurses/clinicians to perform the assessment. Taking all this under consideration, this could allow for a better revival in the healthcare and economic sectors in many countries where COVID-19 is still hitting. In addition, the proposed study uses the raw breathing-only signals as 1D input to deep learning, which when compared to current state-of-art methods that use 2D image transformations, is considered lighter for telemedicine applications with less computational demand. Moreover, majority of studies rely on coughing recordings instead of breathing sounds, which was not the case in this work that depends on regular breathing patterns.

We have elaborated on the performance of the trained deep learning model by extracting the BiLSTM activations. This allows for understanding the machine-based decisions when discriminating between COVID-19 and healthy subjects (**See Results and Fig. 9, Page 12**).

We have further improved the whole manuscript, including the results section, by adding the following,

1) Complete statistical analysis using linear regression fitting for subjects clinical information and MFCC observations for both shallow and deep breathing recordings (**Tables 1, 2, and 3**).

2) A new subsection in the Results section named "**Patient clinical information**" to discuss the observations found when statistically analyzing patient profiles.

3) **Table 4** that shows a summary of the current state of art in COVID-19 detection using respiratory sounds and machine learning relative to the proposed approach.

4) A new subsection in the Results section named "**Performance relative to current state-of-art**" to discuss further the performance relative to literature.

5) Extra figures for shallow breathing signals and MFCC transformation (**Figures 2 and 5**).

6) A complete pseudocode of the proposed study for the development of a trained deep learning model (**Algorithm 1**).

7) Additional information in the Discussion section on the clinical observations found in this study relative to COVID-19 literature "**Section Clinical relevance**".

8) Additional results after the extraction of BiLSTM network activations for COVID-19 and healthy subjects "**Section Neural network activations and Fig. 9**".

9) All algorithms used to re-generate the proposed study including MFCC extraction and deep learning modelling.

We hope that the revised manuscript provides rich information to the readers. We appreciate the concern of this reviewer which have led to pushing the readability and rationale of the proposed work, and we appreciate their re-consideration of our manuscript in its revised form.
