# Supplementary material for: Detection of COVID-19 in smartphone-based breathing recordings: A pre-screening deep learning tool
Source: PLoS One. 2022 Jan 13;17(1):e0262448. doi: 10.1371/journal.pone.0262448 (PMC8758005; doi:10.1371/journal.pone.0262448)
Supplement: Attachment — Submitted filename: Response_to_reviewers.pdf [file pone.0262448.s002.pdf]

REVIEWER 1

In my opinion, the paper is ready for publication. The authors improved the paper to all comments

----

REVIEWER 2

This manuscript is well revised; however, a minor revision is required to improve it further

Concern #1: Deep learning is an important research topic, so how useful for COVID? add some related methods in the related work such as; i) COVID-19 Case Recognition from Chest CT Images by Deep Learning, Entropy-Controlled Firefly Optimization, and Parallel Feature Fusion; ii) Deep Rank-Based Average Pooling Network for Covid-19 Recognition; iii) Screening of COVID-19 Patients Using Deep Learning and IoT Framework.

Response: We appreciate the re-consideration of our manuscript by this reviewer. Accordingly, we have added the suggested works to the introduction and literature review of the revised manuscript (**See Section Introduction, Page 2**).

Concern #2: The related work section should be improved. i) Pseudo Zernike Moment and Deep Stacked Sparse Autoencoder for COVID-19 Diagnosis; ii) COVID19 Classification Using CT Images Via Ensembles of Deep Learning Models; iii) Prediction of COVID-19-pneumonia based on selected deep features and one class kernel extreme learning machine; iv) A novel framework for rapid diagnosis of COVID-19 on computed tomography scans

Response: We have added the suggested works to the introduction and literature review of the revised manuscript (**See Section Introduction, Page 2**).

Concern #3: Algorithm 1 should be written in the form of proper algorithm like mathematical

Response: We preferred to keep the algorithm simple and easy to follow with less mathematical derivations to prevent confusions when re-doing the study by other researchers in future. However, we have kept all mathematical equations and derivations of features in text with proper indexing within the Algorithm.

Concern #4: What are the difference here among spectral and simple entropy

Response: Briefly, in sample entropy, the signal is analyzed in time and phase domains. On the other hand, spectral entropy analyzes the frequency spectrum of the signal. Therefore, we end up

by extracting features from time and frequency domains. We have added more information to the descriptions of both features in the revised manuscript (**See Section Hand-crafted features, page 7**).

Concern #5: How deep activated features are computed? add some point to point description

Response: We compute deep activated features from the last layer of the network, which is the BiLSTM. These features are learned by the network as the second part in the network after the extracted CNN features. To compute these activations, we use function ***activations()*** in MATLAB inputting the trained network, the selected data (breathing recording), and the chosen features layer (BiLSTM). More information was added to the manuscript (**See Section BiLSTM activations, page 9**).

Concern #6: No need of eq. 13-17, these measures are well known. just add the name and ref

Response: We have updated the revised manuscript accordingly by removing the equations and adding a suitable reference (**See Section Performance evaluation, page 10**)

Concern #7: The comparison is not added. If you are not interested to add comparison then add a statistical analysis. You can refer the following work: i) Intelligent Fusion-Assisted Skin Lesion Localization and Classification for Smart Healthcare ii) A two-stream deep neural network-based intelligent system for complex skin cancer types classification

Response: We provided a complete comparison table of our dataset, methodology, and performance with other research works in the same field (AI and breathing sounds) after the suggestions of the first reviewer (**See Table 4, Section Performance relative to current state-of-art, page 14**). The table shows the performance of using shallow breathing and deep breathing recordings (accuracy in %) versus current state-of-art studies. In addition, the comparison between shallow breathing and deep breathing recordings are provided in **Figures 7 and 8**. We hope that the inclusion of the comparison table and figures satisfies this concern for the reviewer.
